# Support Loss and *Q* Factor Enhancement for a Rocking Mass Microgyroscope

**DOI:** 10.3390/s111009807

**Published:** 2011-10-19

**Authors:** Xiong Wang, Dingbang Xiao, Zelong Zhou, Xuezhong Wu, Zhihua Chen, Shengyi Li

**Affiliations:** School of Mechanical Engineering and Automation, National University of Defense Technology, Changsha, Hunan 410073, China; E-Mails: wangxiong0081@sina.com (X.W.); zhouzelong2005@163.com (Z.Z.); xzwu@nudt.edu.cn (X.W.); czhnudt804@163.com (Z.C.); syli@nudt.edu.cn (S.L.)

**Keywords:** rocking mass gyroscope, microgyroscope, support loss, *Q* factor

## Abstract

A rocking mass gyroscope (RMG) is a kind of vibrating mass gyroscope with high sensitivity, whose driving mode and sensing mode are completely uniform. MEMS RMG devices are a research hotspot now because they have the potential to be used in space applications. Support loss is the dominant energy loss mechanism influencing their high sensitivity. An accurate analytical model of support loss for RMGs is presented to enhance their *Q* factors. The anchor type and support loss mechanism of an RMG are analyzed. Firstly, the support loads, powers flowing into support structure, and vibration energy of an RMG are all developed. Then the analytical model of support loss for the RMG is developed, and its sensitivities to the main structural parameters are also analyzed. High-*Q* design guidelines for rocking mass microgyroscopes are deduced. Finally, the analytical model is validated by the experimental data and the data from the existing literature. The thicknesses of the prototypes are reduced from 240 μm to 60 μm, while *Q* factors increase from less than 150 to more than 800. The derived model is general and applicable to various beam resonators, providing significant insight to the design of high-*Q* MEMS devices.

## 1. Introduction

A rocking mass gyroscope is a kind of dual-axial symmetric vibrating mass gyroscope, consisting of four slender beams attached to a rocking mass post in the middle [1]. The driving mode and sensing mode are two perpendicular degenerate modes of the axially symmetric body; the two operational modes are completely uniform, and their natural frequencies are equal. In practice, there always exists a resonance frequency difference between the two operational modes due to asymmetries introduced in fabrication, even if it is much less than 1 Hz [2,3]. MEMS RMG devices have the potential to be microgyroscopes with high sensitivity, which is hoped will be used in space applications [4]. Rocking mass microgyroscopes and other rocking mass devices are now a research hotspot [5–8].

Another important factor, influencing the high sensitivity of RMG, is its *Q* factor. For a microgyroscope operating in air, several energy loss mechanisms coexist. The measured *Q* factor includes those mechanisms, such as air damping loss *Q_air_*, support loss *Q_support_*, thermoelastic damping loss *Q_ted_*, surface loss *Q_surface_*, and the remaining damping effects *Q_other_* [9]. For a microgyroscope operating in vacuum, air damping loss *Q_air_* can be omitted. The measured *Q* factor is mainly the combination of those mechanisms by the expression:

1 $$\frac{1}{Q}=\frac{1}{Q_{\mathrm{air}}}+\frac{1}{Q_{\mathrm{support}}}+\frac{1}{Q_{\mathrm{ted}}}+\frac{1}{Q_{\mathrm{suface}}}+\frac{1}{Q_{\mathrm{other}}}$$

*Q_ted_* is reported to limit the *Q* factor of vacuum packaged microgyroscopes to values ranging from 100,000 to 200,000, while *Q_surface_* is negligible due to the large surface-to-volume ratio of rocking mass gyroscopes [10,11]. *Q_other_* captures the remaining damping effects estimated around 250,000 [12]. *Q_support_* is due to support loss which could be lower than 10,000 depending on the anchor types and materials [13]. *Q_support_* is considered as the dominant energy loss mechanism, and there are several anchor types. Hao presented analytical models for support loss in micromachined beam resonators, only with in-plane flexural vibrations [11,14]. Judge provided analytical models of support loss for MEMS and NEMS beam resonators with out-of-plane flexural vibrations, within the limits of thick and thin support: semi-infinite solid and finite-thickness plate models [15]. Chouvion developed models to predict vibration transmission and support loss in ring-based MEMS sensors based on Judge’s model [16]. Support loss in similar rocking mass resonator has been studied in [17]. This paper will study mainly the effects of the rocking mass post on support loss for the microgyroscope.

This paper thus aims to provide an accurate analytical model of support loss for an RMG to enhance its *Q* factor. The anchor type and support loss mechanism of RMG are analyzed. Support loss is simplified as a model with a beam attached to a support plate at its end. The support loads, power flowing into the support structure, and the vibration energy of RMG are all developed. The analytical model of support loss for RMG is developed, and its sensitivities to the main structural parameters are analyzed. High-*Q* design guidelines for rocking mass microgyroscope are also deduced. The analytical model is validated by the experimental data and the data from the existing literature.

## 2. The Support Loss Mechanism for RMGs

### 2.1. The Anchor Type Analysis of RMGs

Support loss depends mainly on the anchor types and materials. The energy lost from micro resonators into the support structure is summarized by three cases: the first case that acts as semi-infinite elastic medium with effectively infinite thickness, the second case that is treated as a plate with in-plane flexural vibrations, and the last case that can be treated as a plate with out-of-plane flexural vibrations. The first case, which is applicable to NEMS resonators, can be solved by modeling as a semi-infinite elastic medium with loads applied at a single point on surface of the half space [15]. Other cases are applicable to most MEMS resonators. The second case can be solved by modeling as a 2-D problem and using a plane strain method [11]. The last case is a 3-D problem. The energy lost into the surrounding structure consists of the net work done by the MEMS resonator at the attachment point.

**Figure 1.**
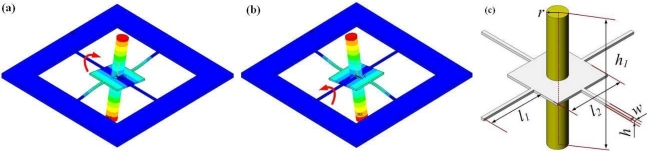
The two operational modes and the main structural parameters of RMG. **(a)** The driving mode. **(b)** The sensing mode. **(c)** The main structural parameters.

The operational modes of RMG are simulated as shown in Figure 1(a,b), which are the two uniform rocking modes, and its main structural parameters are shown in Figure 1(c). When RMG vibrates in its rocking modes, two beams vibrate in line as out-of-plane flexural vibrations, while the other two perpendicular beams vibrate as torsion vibrations at the same time; viz., the four beams are undergoing coupled bending and torsion vibrations.

The dimensions of the attachment point are small compared with the vibration wavelength in the substrate at the natural frequency of RMG. So the support case of an RMG can be treated as a 3-D problem with a finite thickness plate. For the case of a support with finite thickness, we consider a semi-infinite plate with a thickness that need not be the same as that of the vibrating structure itself. Using the plate-edge admittance results [18], we derive analytical expressions of support loss, which also are applicable to a variety of resonators.

### 2.2. Support Loss Mechanism for a RMG

**Figure 2.**
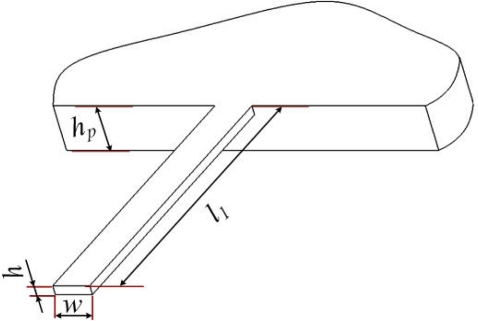
The support model and its main structural parameters.

The power lost from a RMG flows mainly into the support structure. Support loss for the RMG can be simplified as a model with a beam attached to the rigid support structure at its end. The model and its main structural parameters are shown in Figure 2, where *l*_1_, *w* and *h* are the length, width and thickness of the beam model, respectively and *h_p_* is the thickness of its support structure. In an ideal situation, the rocking mass post can be seen as a rigid body without distortion, which is used to transfer kinetic energy without energy loss. When the microgyroscope vibrates at the original position, the total energy is translated into the kinetic energy of the beams and the rocking mass post.

In such cases, the effects of the microgyroscope on its substrate can be modeled as harmonic point forces and moments acting at the attachment point. An assumption is given: the energy propagated into the support structure would not be reflected, viz., all energy that reaches the support is considered lost.

The estimated *Q* factor is thus a lower bound. The *Q* factor is the ratio of the vibration energy of RMG to the energy lost; the reciprocal of *Q* is loss factor *δ* [19]:

(2) $$\delta =\frac{1}{Q}=\frac{\Delta U}{2\pi U}=\frac{\Pi }{\omega U}$$

where Δ*U* is the total energy lost per cycle of oscillation, due to all applicable loss mechanisms, Δ*U = 2π*Π/*ω. U* is the total vibration energy of oscillation. Π is the total net power flow out of RMG, and the average power is simply, Π = 1/2 *Re* (*F*·*V*). *F* is a vector of the point loads, and *V* is the corresponding vector of the harmonic linear and angular velocities at that point.

## 3. The Coupled Vibration Analysis of a RMG

### 3.1. Support Loads Analysis of a RMG

**Figure 3.**
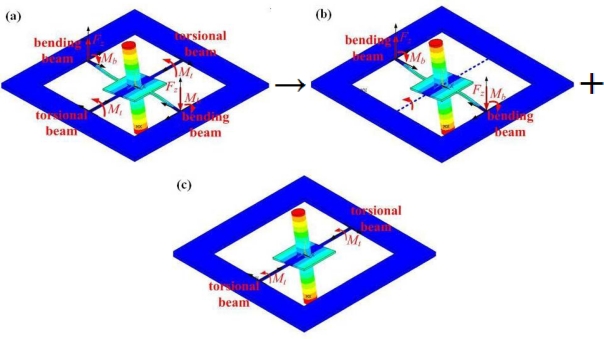
The support loads of RMG and the equivalent SDOF system. **(a)** Attached loads of RMG. **(b)** rocking mode of SDOF system. **(c)** torsional mode of SDOF system.

The operational modes of a RMG can be considered equivalent to a superposition of the rocking mode and torsional mode of an equivalent single degree of freedom (SDOF) system, which has the rocking mass post and two beams in line. The loads of the attachment points are described in Figure 3, without considering gravitation. There are two loads at the end of each bending beam, viz., the shear force normal to the substrate *F_z_*, and the bending moment about the axis parallel to the substrate edge *M_b_*. There is only one torsional moment about the axis perpendicular to the substrate edge *M_t_*, at the end of each torsional beam. When a RMG vibrates in its operational modes, the four beams vibrate as a coupled bending and torsion. The arbitrary amplitude of the bending angle and the torsional angle at the end of these beams are equal, denoted by *θ*_0_.

Using micro scanners’ vibration mode frequency results [20], these loads scaled by *θ_0_* and the spring constant of RMG can be expressed, respectively as:

(3) $$M_{t}={\mathrm{GI}}_{p}\theta_{0}/l_{1}$$

(4) $$M_{b}=-{\mathrm{EI}}_{y}\;\left(4l_{1}+3l_{2}\right)\theta_{0}/l_{1}^{2}$$

(5) $$F_{z}=-6{\mathrm{EI}}_{y}\;\left(l_{1}+l_{2}\right)\theta_{0}/l_{1}^{3}$$

(6) $$K=\frac{2{\mathrm{GI}}_{p}}{l_{1}}+\frac{{\mathrm{Ewh}}^{3}}{l_{1}}\left[\frac{1}{2}{\left(\frac{l_{2}}{l_{1}}\right)}^{2}+\frac{l_{2}}{l_{1}}+\frac{2}{3}\right]$$

where *E* is Young’s modulus, *G* is shear modulus, *ρ* is density. *h* is the thickness of the vibratory structure, *l*_1_ is the length of the beams, *l*_2_ is the length of the center support. *I_p_* is the polar moment of inertia of the beams’ cross-section, *I_y_* is the moment of inertia of the beams’ cross-section, *K* is the spring constant of the rocking vibration.

### 3.2. Power Flow into the Support Structure

**Figure 4.**
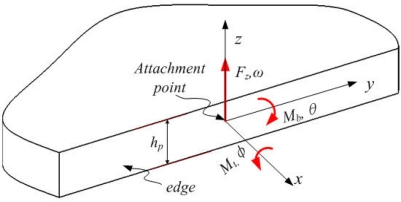
Semi-infinite plate and the applied loads to attachment point.

The energy loss can be found by considering the plate support responding to the loads applied to its edge because the support structure is modeled as a plate. Consider the shear force *F_z_*, the bending moment *M_b_*, and the torsional moment *M_t_*, as shown in Figure 4. For RMG, the force normal to the plate edge, the shear force parallel to the plate, and the bending moment about the axis perpendicular to the plate are not considered. The admittance at the edge of a plate was first formulated in integral form by Eichler. The elements of the matrix Y are given as closed-form integrals [21]. These integrals have recently been solved in closed form [22].

The point mobility matrix Y relates the normal angular velocity *Ω_b_*, the tangential angular velocity *Ω_t_*, and the transverse linear velocity *V_z_*, of the attachment point to these applied loads by the expression:

(7) $$\left[\begin{array}{c} \Omega_{b}\\ \Omega_{t}\\ V_{z} \end{array}\right]=i\omega \left[\begin{array}{c} \theta \\ \phi \\ w \end{array}\right]=\frac{1}{\sqrt{\rho_{p}h_{p}D}}\left[Y\right]\left[\begin{array}{c} M_{b}\\ M_{t}\\ F_{z} \end{array}\right]=\frac{1}{\sqrt{\rho_{p}h_{p}D}}\left[\begin{array}{ccc} y_{11}k^{2} & 0 & 0\\ 0 & y_{22}k^{2} & y_{23}k\\ 0 & y_{32}k & y_{33} \end{array}\right]\left[\begin{array}{c} M_{b}\\ M_{t}\\ F_{z} \end{array}\right]$$

where *D* is the plate stiffness, *D* = *Eh_p_*^3^/12(1 − *υ*^2^), *h_p_* is its thickness, *k* is the free wave numbers in the solid at frequency *ω*, *k*= [*ω*(*ρh_p_*/*D*)^1/2^]^1/2^. The corresponding coefficients have been calculated, for *υ* = 0.3, which were calculated by Su [22]; for *υ* = 0.28, which were calculated by Chouvion [16], as shown in Table 1. The material in our experiments is n-type (100) single crystal silicon, Poisson ratio *υ* = 0.28, so these coefficients can be determined.

The resulting expressions for the power radiated into the plate support are:

(8) $$\Pi_{F_{z}}=\sqrt{3\left(1-\upsilon^{2}\right)}y_{33}\frac{F_{z}^{2}}{h_{p}^{2}\sqrt{E\rho }}$$

(9) $$\Pi_{M_{b}}=6\;\left(1-\upsilon^{2}\right)y_{11}\frac{\omega M_{b}^{2}}{{\mathrm{Eh}}_{p}^{3}}$$

(10) $$\Pi_{M_{t}}=6\;\left(1-\upsilon^{2}\right)y_{22}\frac{\omega M_{t}^{2}}{{\mathrm{Eh}}_{p}^{3}}$$

(11) $$\Pi_{M_{b}\;\mathrm{and}\;F_{z}}=\Pi_{F_{z}}+\Pi_{M_{b}}$$

(12) $$\Pi_{M_{b}\;\mathrm{and}\;M_{t}}=\Pi_{M_{b}}+\Pi_{M_{t}}$$

(13) $$\Pi_{M_{t}\;\mathrm{and}\;F_{z}}=\Pi_{M_{t}}+\Pi_{F_{z}}+{\left[12\left(1-\upsilon^{2}\right)\right]}^{3/4}y_{23}\frac{\omega^{1/2}M_{t}F_{z}}{\rho^{1/4}E^{3/4}h_{p}^{2/5}}$$

When each load is considered individually, the Equations (8–10) apply for the isolated load conditions, while the Equations (11–13) apply for two coupled load conditions. Equation (11) is different from Equation (13) presented by Judge [15]. The reason is that its velocity expressions are wrong compared with Equation (4) presented by Su [22]. However, the off-diagonal terms of Y result in an additional contribution that the total power is in Equation (13) if both the torsional moment and the shear force are present.

### 3.3. The Effective Inertia and Stored Kinetic Energy of RMG

Using Raleigh’s method, the kinetic energy stored and the effective inertia of the bending beams can be expressed, respectively as [20]:

(14) $$U_{b}={\mathrm{KE}}_{b}=\int_{0}^{l_{1}}\frac{1}{2}\left(\rho \mathrm{wh}\right)\;{\left(\frac{d\omega \left(x\right)}{\mathrm{dt}}\right)}^{2}\mathrm{dx}=\frac{1}{2}\rho {\mathrm{whl}}_{1}\;\left(\frac{1}{105}l_{1}^{2}+\frac{5.5}{105}l_{1}l_{2}+\frac{9.75}{105}l_{2}^{2}\right){\left(\frac{d\theta }{\mathrm{dt}}\right)}^{2}$$

(15) $$J_{b}=\rho {\mathrm{whl}}_{1}\;\left(\frac{1}{105}l_{1}^{2}+\frac{5.5}{105}l_{1}l_{2}+\frac{9.75}{105}l_{2}^{2}\right)$$

Then, the kinetic energy and effective inertia of the torsion beams and rocking mass post are also solved:

(16) $$U_{t}={\mathrm{KE}}_{t}=\int_{0}^{l_{1}}\frac{1}{12}\rho \mathrm{wh}\;\left(w^{2}+h^{2}\right)\;{\left(\frac{x}{l_{1}}\frac{d\theta }{\mathrm{dt}}\right)}^{2}\mathrm{dx}=\frac{1}{36}\rho {\mathrm{whl}}_{1}\;\left(w^{2}+h^{2}\right)\;{\left(\frac{d\theta }{\mathrm{dt}}\right)}^{2}$$

(17) $$J_{t}=\frac{1}{18}{\rho \mathrm{whl}}_{1}\;\left(w^{2}+h^{2}\right)$$

(18) $$U_{m}={\mathrm{KE}}_{m}=\frac{1}{2}J_{m}\;{\left(\frac{d\theta }{\mathrm{dt}}\right)}^{2}$$

(19) $$J_{m}=\frac{1}{12}M_{m1}\;\left(l_{2}^{2}+h^{2}\right)+\frac{1}{12}M_{m2}\;\left(\frac{3}{4}D^{2}+h_{1}^{2}\right)$$

where *θ* is the rocking angle of the rocking mass post, *θ = θ*_0_ sin(*ωt*), *dθ/dt* = *ωθ*_0_ cos(*ωt*). *M*_*m*1_ is the mass of the center support, and *M*_*m*2_ is the mass of the rocking mass post. The formulae of the vibratory energy are also scaled by *θ*_0_, the arbitrary amplitude of the rocking vibration mode shape.

## 4. Support Loss Prediction of a RMG

### 4.1. Support Loss Prediction

The vibration energy and the resonant frequency for the operational modes of RMG are expressed, respectively as:

(20) $$U_{\mathrm{total}}=U_{m}+2U_{b}+2U_{t}=\frac{1}{2}J_{\mathrm{xx}}\omega^{2}\theta_{0}^{2}$$

(21) $$\omega =\sqrt{k/J_{\mathrm{xx}}}$$

where the total effective inertia of RMG can be solved as *J_xx_ = J_yy_ = J_m_ + 2J_b_ + 2J_t_*.

Given *w* ≥ *h*, *I_p_* *= βwh*^3^, *β* is the function of *w*/*h* [23]. The radiated power can be found by substituting Equations (10,11,20,21) into Equation (2), and the support loss factor is found as Equation (22); Given *w* < *h*, the support loss factor is found as Equation (23):

(22) $$\frac{1}{Q}=\frac{\sqrt{3\left(1-\nu^{2}\right)J_{\mathrm{xx}}}y_{33}{\left(l_{1}+l_{2}\right)}^{2}}{{\left(\frac{\beta }{1+\nu }+\eta \right)}^{3/2}l_{1}^{4}\sqrt{\rho h}}\frac{\sqrt{w}}{\sqrt{l_{1}}}\frac{h^{2}}{h_{p}^{2}}+\left[\frac{\left(1-\upsilon^{2}\right)y_{11}{\left(4l_{1}+3l_{2}\right)}^{2}}{6\left(\frac{\beta }{1+\nu }+\eta \right)l_{1}^{2}}+\frac{6\left(1-\upsilon \right)y_{22}\beta^{2}}{\left(1+\nu \right)\left(\frac{\beta }{1+\nu }+\eta \right)}\right]\frac{w}{l_{1}}\frac{h^{3}}{h_{p}^{3}}$$

(23) $$\frac{1}{Q}=\frac{\sqrt{3\left(1-\nu^{2}\right)J_{\mathrm{xx}}}y_{33}{\left(l_{1}+l_{2}\right)}^{2}}{{\left(\frac{\beta }{1+\nu }\frac{w^{2}}{h^{2}}+\eta \right)}^{3/2}l_{1}^{4}\sqrt{h\rho }}\frac{\sqrt{w}}{\sqrt{l_{1}}}\frac{h^{2}}{h_{p}^{2}}+\left[\frac{\left(1-\upsilon^{2}\right)y_{11}{\left(4l_{1}+3l_{2}\right)}^{2}}{6\left(\frac{\beta }{1+\nu }\frac{w^{2}}{h^{2}}+\eta \right)l_{1}^{2}}+\frac{6\left(1-\upsilon \right)y_{22}\beta^{2}}{\left(1+\nu \right)\left(\frac{\beta }{1+\nu }+\frac{h^{2}}{w^{2}}\eta \right)}\right]\frac{h}{l_{1}}\frac{w^{3}}{h_{p}^{3}}$$

where *η* = (*l*_2_/*l*_1_)^2^/2 *+ l*_2_/*l*_1_ + 2/3. It can be seen from Equations (22) and (23) that, for *h_p_* is large relative to *h*, the shear force term *F_z_* dominates the support loss, and the effect of the bending moment *M_b_* and the torsional moment *M_t_* at the attachment point could be neglected.

**Figure 5.**
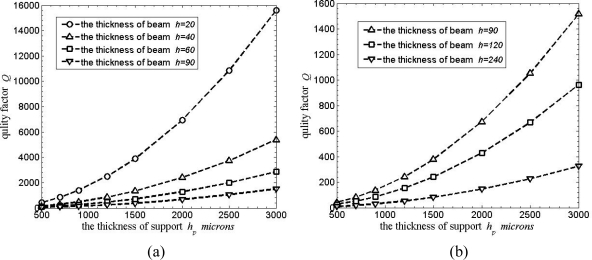
Support loss for rocking mass gyroscope. **(a)** *Q* factor dependent on *h_p_* (*w* ≥ *h*). **(b)** *Q* factor dependent on *h_p_* (*w* < *h*).

For *ν* = 0.28, *w* = 90, *l*_1_ = 2,200, *l*_2_ = 2,200, *h*_1_ = 5,000, *r* = 550. Support loss for the rocking modes in RMG is shown in Figure 5. When *w* ≥ *h*, four curves are shown relating *Q* factor to the thickness of the support plate *h_p_* in Figure 5(a), while *w* < *h*, three curves are shown in Figure 5(b). *Q* factor increases with the thickness of the support *h_p_* increasing; furthermore, the thickness of the beams *h* is thinner, *Q* factor will increase more obviously. For *h* = 90 μm, *Q* factor increases from almost 50 to 1,600 when the thickness *h_p_* increases from 500 to 3,000 μm; while for the 20 μm thick beams, *Q* factor can reach almost 16,000. Compared with the predicted results in [17], these results are almost an order of magnitude less than those without considering the rocking mass post, viz., the rocking mass post would badly influence Q factor of the microgyroscope. These curves provide a good order-of-magnitude estimate of the support loss for the microgyroscope.

### 4.2. Parameter Sensitivity Analysis

In this section, the sensitivities of support loss to the main structural parameters involved, such as sizes of the rocking mass post, center support, and beams are studied.

**Figure 6.**
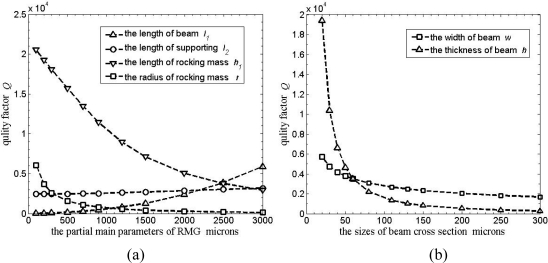
Support loss for rocking mass gyroscope. **(a)** *Q* dependent on partial main parameters. **(b)** *Q* dependent on the cross section sizes of the beams.

A plot of support loss for *ν* = 0.28, *w* = 90, *h* = 60, *h_p_* = 2,000 is shown in Figure 6(a). Four curves show relating *Q* factor to the length *l*_1_, *l*_2_, *h*_1_ and the radius *r*. *Q* factor increases with the length *l*_1_ and *l*_2_ increasing; *l*_1_ dominates the increasing of *Q* factor, and *l*_1_ must be large enough to achieve high *Q* factor. *Q* factor decreases with the length *h*_1_ and the radius *r* increasing quickly, and *r* must be small enough to achieve high *Q* factor and larger natural frequency.

Furthermore, the thickness *h* and width *w* of the beams are also studied. For *ν* = 0.28, *h_p_* = 2,000, *l*_1_ = 2,200, *l*_2_ = 2,220, *r* = 500, *h*_1_ = 3,000, a plot of support loss relating *Q* factor to *h* and *w* is shown in Figure 6(b). Two curves show that *Q* factor decreases quickly with the two parameters increasing; *h* dominates the decreasing of *Q* factor, and *h* must be small enough to achieve high *Q* factor for RMG.

### 4.3. Design Guidelines for High-*Q*

The analytical model derived provides the design guidelines for achieving high *Q* factor in RMG, which may be summarized as below:

1. Choice of materials: in order to increase *Q_support_*, the high-strength material is preferred for the rocking mass post and the substrate (for higher vibration energy and lower energy loss), while the low-strength material for the beams.
2. Geometrical dimensions:
  - *Q_support_* increases with the length of the beams and the center support (*l_1_* and *l_2_*); however, the length of the beams (*l_1_*) dominates *Q* factor, and the comparatively longer beams will increase *Q_support_*, while considering the frequency design.
  - *Q_support_* decreases with the rocking mass post dimensions (*h_1_* and *r*). The dimensions should be chosen with care to improve the sensitivity of RMG, while maximizing *Q_support_*.
  - *Q_support_* is highly sensitive to the thickness ratio of the support structure and the beams ((*h_p_*/*h*)^2^ and (*h_p_*/*h*)^3^) of RMG. The thicker support structure and the slenderer beams will increase *Q_support_*, while considering the structural reliability.

## 5. Experimental Verification and Discussions

**Figure 7.**
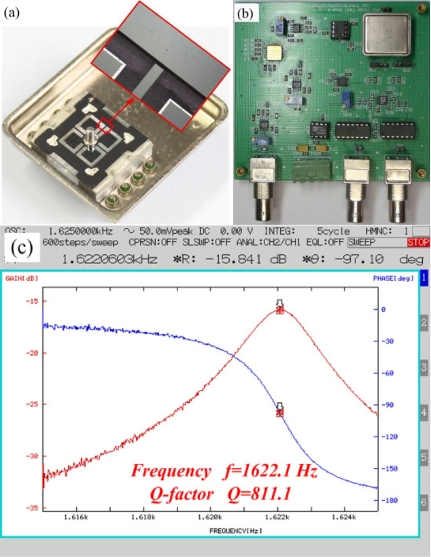
The prototype and support, mode measuring system and frequency response curve. **(a)** The prototype and support. **(b)** Mode measuring board. **(c)** Frequency response of the prototype.

The predicted results of the analytical model are compared with the experimental results to demonstrate validity of the model for RMG. Experiments are conducted on several rocking mass microgyroscope prototypes. The prototypes are fabricated by using same silicon wafers and the same structural parameters, except for the different thicknesses of the vibratory structure changed by a thickness reducing technology. The electrostatic actuation and capacitance detection method is used to measure the natural frequencies and *Q* factor. The prototypes are designed with a part at each corner of the center support to be measured conveniently, as shown in Figure 7(a). Measurements of the natural frequencies and *Q* factor are obtained by sweeping frequency of harmonic drive signal and using half-power bandwidth method. The measuring setup is composed of the vacuum chamber, DC power, Agillent 33250A waveform generator, frequency response analyzer FRA 5087, and mode measuring board. The mode measuring board and the prototypes, as shown in Figure 7(b), are put into the vacuum chamber. Then measurements are conducted, and a frequency response curve of the measured prototypes is shown in Figure 7(c).

All the measured results, which are measured at a lower pressure of less than 10 Pa in the vacuum chamber, are compared with the theoretical predictions in Table 2. These results are measured and calculated by using the half-power bandwidth method, and they are referenced values for the accuracy. The thickness of the support in our experiments is estimated as 2,500 ìm, and all the predicted values of *Q* factor in Table 2 correspond to the calculated values in Figure 5. The measured results are lower than the predictions in all cases, but show the same general trend as the predicted values of *Q_total_*. These results indicate that a significant amount of the vibration energy is radiated into the support structure. Note that it is not expected that the data should fall directly on the theoretical values, since the measurement is the total *Q*, including the contributions of support loss, thermoelastic damping loss, surface loss and other losses. The calculated results thus represent an upper bound that the measured data would be expected to approach when other loss mechanisms are negligible.

Besides, the silicon structure and the Pyrex base plate are bonded together by coating epoxy resin. The poor coating uniformity will induce serious energy losses. Some Q values are thus much smaller than other Q values of the prototypes with the same dimensions and in the same batch, and 2# prototype is one of the cases. Compared with the experimental results in [17], the presented results in Table 2 are almost an order of magnitude less than the formers.

Bae presented some measurements of *Q* factor for JPL’s rocking a mass microgyroscope in a ceramic substrate package [2]. The gyroscopes attached to a substrate more than 3,000 μm thick, which is still only a small fraction of the shear wavelength at the resonant frequencies of its operational modes, although over 115 times thicker than the 26 μm thick vibratory beams. The appropriate model for the support is thus the plate model, *i.e.*, the power flow into the support is obtained via Equation (20), and the loss factor is obtained via Equation (22). The *Q* values of JPL’s microgyroscope were measured via the ring-down time method, and a mean *Q* value of 28,000 was achieved, which was of the similar order of magnitude of the prediction in Equation (22).

By comparing all the experimental results, and to comment on their utility in various thicknesses of the microgyroscopes, we conclude that all the cases indicated that the dominant loss mechanism for RMG may be the radiation into the support structure. The expressions for the power flow, presented in Section 3.2, are applicable for any other resonator geometries for which the attachment to the support structure acts essentially as a point source for vibration in the support.

## 6. Conclusions

An accurate analytical model of support loss for a RMG is presented. The anchor types and the support loss mechanism of the RMG are firstly analyzed, and the support loss is simplified as a model with a beam attached to the support plate at its end. The support loads of the RMG are analyzed, and then the powers flowing into the support and the vibration energy of the RMG are also derived. The analytical model of the support loss for RMG is developed, and its sensitivities of the support loss to the main structural parameters are analyzed. Finally, the high-*Q* design guidelines for rocking mass microgyroscope are given. The analytical model is validated by the experimental data and the data from the existing literature. The thicknesses of the prototypes are reduced from 240 μm to 60 μm, while *Q* factor increases from less than 150 to more than 800. Compared with both the predicted results and the experimental results, the presented results in this paper are almost an order of magnitude less than those without considering the rocking mass post. The derived model is general and applicable to various beam resonators, providing significant insight into the design of high-*Q* MEMS devices.

## Figures and Tables

**Table 1.** The numerical values of coefficients for Y.

**coefficients**	***υ* = 0.30**	***υ* = 0.28**
Re(y_11_) = Re(y_22_)	0.21645	0.22172
Re(y_23_) = Re(y_32_)	−0.29149	−0.28546
Re(y_33_)	0.46198	0.45735

**Table 2.** The predicted *f* and *Q*, measured *f* and *Q* for rocking mass gyroscope prototypes.

**Gyro#**	***h/h_p_* (μm)**	**Predicted *f* (Hz)**	**Measured *f* (Hz)**		**Predicted *Q***	**Measured *Q***	
			***f*_1_**	***f*_2_**		***Q*_1_**	***Q*_2_**
2#	240/2,500		5,760.0	5,755.4		76.8	78.8
4#	240/2,500	5,853.4	5,683.2	5,678.6	226.8	123.3	125.6
5#	240/2,500		5,858.8	5,842.8		128.4	135.9
3#	120/2,500		2,697.9	2,706.2		355.0	337.4
4#	120/2,500	2,996.4	2,768.9	2,778.1	668.6	325.4	321.9
7#	120/2,500		2,760.5	2,756.2		270.4	256.9
01#	60/2,500		1,726.2	1,799.8		722.8	789.4
06#	60/2,500	1,828.1	1,707.0	1,793.2	1,992.0	742.2	674.1
11#	60/2,500		1,622.1	1,689.2		811.1	796.8
